# Visual working memory and sensory processing in autistic children

**DOI:** 10.1038/s41598-021-82777-1

**Published:** 2021-02-11

**Authors:** Ryan A. Stevenson, Justin Ruppel, Sol Z. Sun, Magali Segers, Busisiwe L. Zapparoli, James M. Bebko, Morgan D. Barense, Susanne Ferber

**Affiliations:** 1 Department of Psychology, University of Western Ontario, Western Interdisciplinary Research Building, 1151 Richmond Street, London, ON N6G 2K3 Canada; 2 Brain and Mind Institute, University of Western Ontario, London, ON Canada; 3 Department of Psychiatry, Schulich School of Medicine and Dentistry, University of Western Ontario, London, ON Canada; 4 Schulich School of Medicine and Dentistry, Neuroscience Program, University of Western Ontario, London, ON Canada; 5 Centre for Vision Research, York University, Toronto, ON Canada; 6 Department of Psychology, University of Toronto, Toronto, ON Canada; 7 Department of Psychology, York University, Toronto, ON Canada; 8 Rotman Research Institute, Toronto, ON Canada

**Keywords:** Psychology, Neurodevelopmental disorders

## Abstract

While atypical sensory processing is one of the more ubiquitous symptoms in autism spectrum disorder, the exact nature of these sensory issues remains unclear, with different studies showing either enhanced or deficient sensory processing. Using a well-established continuous cued-recall task that assesses visual working memory, the current study provides novel evidence reconciling these apparently discrepant findings. Autistic children exhibited perceptual advantages in both likelihood of recall and recall precision relative to their typically-developed peers. When autistic children did make errors, however, they showed a higher probability of erroneously binding a given colour with the incorrect spatial location. These data align with neural-architecture models for feature binding in visual working memory, suggesting that atypical population-level neural noise in the report dimension (colour) and cue dimension (spatial location) may drive both the increase in probability of recall and precision of colour recall as well as the increase in proportion of binding errors when making an error, respectively. These changes are likely to impact core symptomatology associated with autism, as perceptual binding and working memory play significant roles in higher-order tasks, such as communication.

## Introduction

The rising prevalence of autism spectrum disorder (ASD) to 1 in every 59 births^1^ and the rising global cost of care^2^ necessitate a better understanding of the etiology and associated heterogeneous symptomology of the condition. Historically, theories explaining ASD focus on disturbances in high-level cognitive functioning such as theory of mind^3^, weak central coherence^4^, or executive dysfunction^5^. More recent accounts, however, have focused on low-level, underlying neurobiological differences that unfold throughout development and cascade into social and cognitive challenges. These include changes in excitation and inhibition spurred by imbalances in GABAergic and glutamatergic synapses^6^, irregular temporal fidelity of neuronal coupling^7^, or deficits in Bayesian statistical learning^8^. These accounts share a common link in providing explanations for atypical sensory perception in autism^9^, which have been both theorized to^10,11^ and empirically shown to impact symptomatology^12^.

Sensory issues are nearly ubiquitous in ASD, affecting up to 94% of autistic individuals^13^. Atypical sensory perception impacts core diagnostic criteria, including social communication and restricted interests and repetitive behaviors^14^. Although sensory issues are commonly discussed as an impairment, there are also areas where autistic individuals outperform their typically-developed (TD) peers^15^. Thus, a complete account of sensory issues in autism must explain not only the observed weaknesses, but also the areas of enhanced performance. To this aim, we employed a well-characterized continuous cued-recall task^16,17^ to study the strengths and weaknesses of maintaining sensory representations in visual working memory (VWM), a mental workspace to connect inputs with higher-order cognition^18^. Participants study a varying number of coloured squares (set size; 2–3 in the current study) in discrete locations and after a brief delay, they report the remembered color of a randomly cued square, indicated by a location probe (i.e., “what colour did you study in this location previously?”). This response is made by selecting the remembered colour from a continuous colour wheel, allowing for the measurement of not only VWM capacity, but also of recall precision, and perceptual binding of colour and location. Autistic individuals often exhibit enhanced visual perceptual abilities on simple visual tasks, such as change detection, colour discrimination, and simple visual search tasks for review, see^19^. In the VWM colour wheel task, the precision of an individual’s response (i.e., how closely individuals recall the exact studied color) would tap into this strength. Therefore, we predicted that autistic individuals would exhibit greater VWM precision than their TD peers and may, overall, show fewer errors.

When an error is made, however, the VWM colour-wheel task affords us with the possibility to determine the origin of such errors, forgetting the studied colour or misbinding a studied colour with the incorrect location. Autistic individuals have difficulties binding sensory information into a unified percept^20–22^: they are less susceptible to visual illusions that require integration of multiple component features^23^, and show reduced benefits of binding a speaker’s face with their voice^24–27^. In a colour-wheel VWM task, these *binding errors* would result in recalling a colour that was presented at a non-target location. We predicted that when errors are made, autistic individuals would be more likely to make binding errors than their TD peers. Therefore, this task allows us to simultaneously observe strengths and weaknesses in sensory processing in the same individuals while keeping the task demands unaltered.

## Methods

### Participants

Participants included 51 children (mean age = 12.0 ± 2.8 years) split into two groups, TD and ASD. TD children (N = 30, 8 males, age range 7–16 years old, mean age = 11 years old) had neither an individual or familial diagnosis of ASD, nor any other neurological condition. Autistic participants (N = 21, 17 males, age range 8–17 years old, mean age = 13 years old) had a formal ASD diagnosis by a clinical practitioner familiar with ASD and provided their clinical report. Diagnoses were confirmed through the administration of the Autism Diagnostic Observation Schedule (ADOS-1 or -2^28^) by a research-reliable clinician. To ensure that TD participants were not on the autism spectrum, and to assess symptom severity in ASD, the Autism Quotient (AQ^29^) and Social Responsiveness Scale (SRS^30^) were administered to all participants. The AQ was scored using Likert scoring^31^, with the TD group scoring significantly lower than the ASD group (TD mean score = 51.3 ± 13.9, ASD mean score = 95.3 ± 15.7, *p* = 8.00e^−14^, *t*_(49)_ = 10.28, *d* = 2.39). Likewise on the SRS, the TD group scored significantly lower than the ASD group (TD mean score = 51.0 ± 8.9; ASD mean score = 75.7 ± 11.6, *p* = 9.62e^−10^, *t*_(49)_ = 7.54, *d* = 2.39). Note that a score of 76 and above on the SRS is considered severe, a score between 60 and 75 is considered indicative of so-called “high-functioning” ASD in those with a diagnosis, and 59 or below is considered asymptomatic. Cognitive abilities were measured using the Weschler Abbreviated Scale of Intelligence (WASI-2^32^) 2-scale subtest. TD participants’ scores did not significantly differ from those of ASD participants (TD mean = 102.9 ± 10.8, ASD mean = 107.4 ± 14.9, *p* = 0.23, *t*_(49)_ = 1.59, *d* = 0.34).

All protocols were approved by the research ethics board at the University of Toronto, and all experiments were performed in accordance with relevant guidelines and regulations. Informed consent was obtained from a parent and/or legal guardian. Participants were compensated monetarily for their time and with a small gift.

### Apparatus

Participants were seated in front of a 15-inch laptop screen at a distance of approximately 60 cm. The screen resolution was 1280 × 960 pixels and the refresh rate was 60 Hz. Stimuli were generated and presented using MATLAB (Natick, MA) and the Psychophysics Toolbox 3 extensions^33–35^.

### Stimuli and procedure

**Figure 1:**
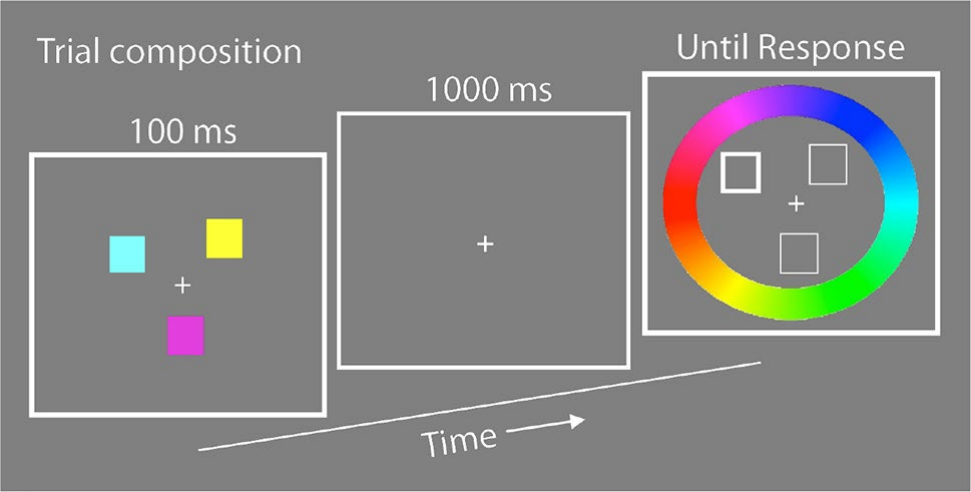
Trial composition. This panel depicts an individual trial (set size = 3). Responses were analyzed with a probabilistic mixture model in which the degree of error between the chosen colour and the actual presented colour is calculated and tabulated across trials.

Stimuli and task design were based on Zhang and Luck^16^ but adapted to be more suitable for a younger age group. Each trial started with a 100 ms long presentation of coloured squares as memory samples, with set sizes of either 2 or 3, around a black fixation cross (1.0° × 1.0° visual angle) on a gray background. The to-be-remembered coloured squares (henceforth, sample items) were 1.2° by 1.2° in size and could be located at 18 possible locations arranged around a virtual circle with a radius of 5.6° (Fig. 1). Participants were instructed to remember as many of the sample items as possible.

Sample item colours were selected from 180 possible colours, evenly distributed along an isoluminant colour wheel (centered at (L = 65 a = 5 b = 5) in the CIEL*a*b* colour space The colours of all sample items on a given trial were randomly chosen without replacement from these 180 colours, with the constraint that two samples colours must be separated by a minimum 15 values (i.e., 40°) in colour space to ensure discriminability.

After a 1000-ms delay screen containing only the fixation cross, the test screen was presented. The locations of the sample items were outlined in black (with no colour fill except the gray background), and the to-be-reported target item’s location bolded to distinguish it from the non-target locations (4 pixel weight for non-targets items, 8 pixel weight for the target item). The test screen also contained a visual presentation of the colour wheel described above, arranged on an annulus with an inner radius of 7.6° and an outer radius of 10.1° The luminance of all possible stimulus colours remained constant and independent of hue.

During the test screen phase, participants used the computer mouse to select the remembered colour of the target square by clicking the location on the colour wheel that most precisely corresponded with the remembered colour of the target item. To facilitate visualization of the chosen colour, when the participant selected a colour from the colour wheel, the target location’s outline was filled with the selected color. Emphasis was placed on precision, and participants were given unlimited time to change and adjust their selection until they were satisfied with their choice. The participants pressed the keyboard space bar when they felt ready to lock in their response and would then advance to the next trial following a 500 ms inter-trial interval. Participants were instructed to avoid selecting non-target colours and instead to guess a random colour if they could not recall the target item. Participants completed 80 trials in total, with 40 trials per set-size condition interleaved within a single block. Prior to data collection, participants completed 10 practice trials to ensure that they understood the task and were completing it properly.

### Analysis

Behavioural data analysis was based on the method outlined by Bays et al. 2008 http://www.bayslab.com^17^. Stimulus colours and participant responses were coded in circular units (from π radians to -π radians), and recall error was calculated as the angular deviation between the actual target colour and the colour selected by the participant.

To investigate participants’ VWM, we employed a probabilistic mixture model developed by Bays et al.^36^. The model assumes that three memory states account for the distribution of responses in a VWM task: recalling the target, recalling a non-target, or recalling nothing from memory which will result in guessing. When the participant can recall the target colour, measured as the probability to recall the target, or *pMem*, target responses are assumed to be normally distributed around the target colour using a circular analogue of the Gaussian distribution, the von Mises distribution. For instances in which the participant retrieves a non-target colour, measured as the probability to recall the non-target, *pNT* or *binding error,* each pNT response is also assumed to be distributed around a non-target colour using a von Mises distributions. Finally, the participant may not be able to access the target at all and has to guess randomly, measured as the probability of guesses, *pGuess*, a uniform distribution around the circle. It should be noted here that pGuess is equal to 1 − (pMem + pNT) and is not considered a parameter itself, thus the parameters pMem and pNT determine the probability of any of the three memory states, all of which must sum to 1.

In addition, the *precision* parameter reflects the fidelity or resolution with which the colour has been represented in memory, when colour can be retrieved (pMem). Recall *precision* was calculated as the reciprocal of the standard deviation of the recall error (using Fisher’s definition of circular standard deviation). Recall precision assesses the degree to which responses cluster around the original target colour (i.e., higher precision scores indicate the participant’s responses cluster more narrowly around the target colour, suggesting more precise responses). It is assumed that the von Mises distribution around target and non-targets are assumed to have the same precision.

Maximum likelihood estimates of the three aforementioned parameters, pMem, pNT, and precision, were separately obtained for each participant and each set-size using an expectation–maximization algorithm. The three memory states defined by these parameters will henceforth be referred to as memory (pMem), binding errors (pNT), and guesses (pGuess), respectively. Model parameter estimation was performed with a range of initial parameter estimates to ensure global maxima were achieved.

Differences between diagnostic groups and set sizes were subsequently tested using a mixed-model analysis of variance (ANOVA) and follow-up t-tests in the parameter values pMem, recall precision, pNT, and pGuess. Also, given significant differences in pMem between groups, the proportion of binding errors relative to all errors, or

$$pNT\left| {Error = pNT/(pNT + pGuess)} \right.$$

were calculated to account for differences in total number of errors, then compared. The proportion of guesses relative to total errors is equal to one minus pNT|Error, thus independent analysis is redundant. Greenhouse–Geisser corrections were applied whenever the sphericity assumption of the ANOVA was violated.

Symptom severity in the ASD group was calculated as the weighted sum of the SRS and AQ measures:

$$Symptom\;Severity = \frac{{{\raise0.7ex\hbox{${AQ_{{score}} }$} \!\mathord{\left/ {\vphantom {{AQ_{{score}} } {AQ_{{\max }} }}}\right.\kern-\nulldelimiterspace} \!\lower0.7ex\hbox{${AQ_{{\max }} }$}} + {\raise0.7ex\hbox{${SRS_{{score}} }$} \!\mathord{\left/ {\vphantom {{SRS_{{score}} } {SRS_{{\max }} }}}\right.\kern-\nulldelimiterspace} \!\lower0.7ex\hbox{${SRS_{{\max }} }$}}}}{2},$$

where AQ_max_ and SRS_max_ represent the maximum score on each measure, resulting in a range of symptom severity from 0 (least severe) to 1 (most severe). The relationship between pMem, precision, and pNT|Error on symptom severity was analyzed via multiple regression.

## Results

### Analysis of target responses: probability of recall (pMem) and precision

**Figure 2:**
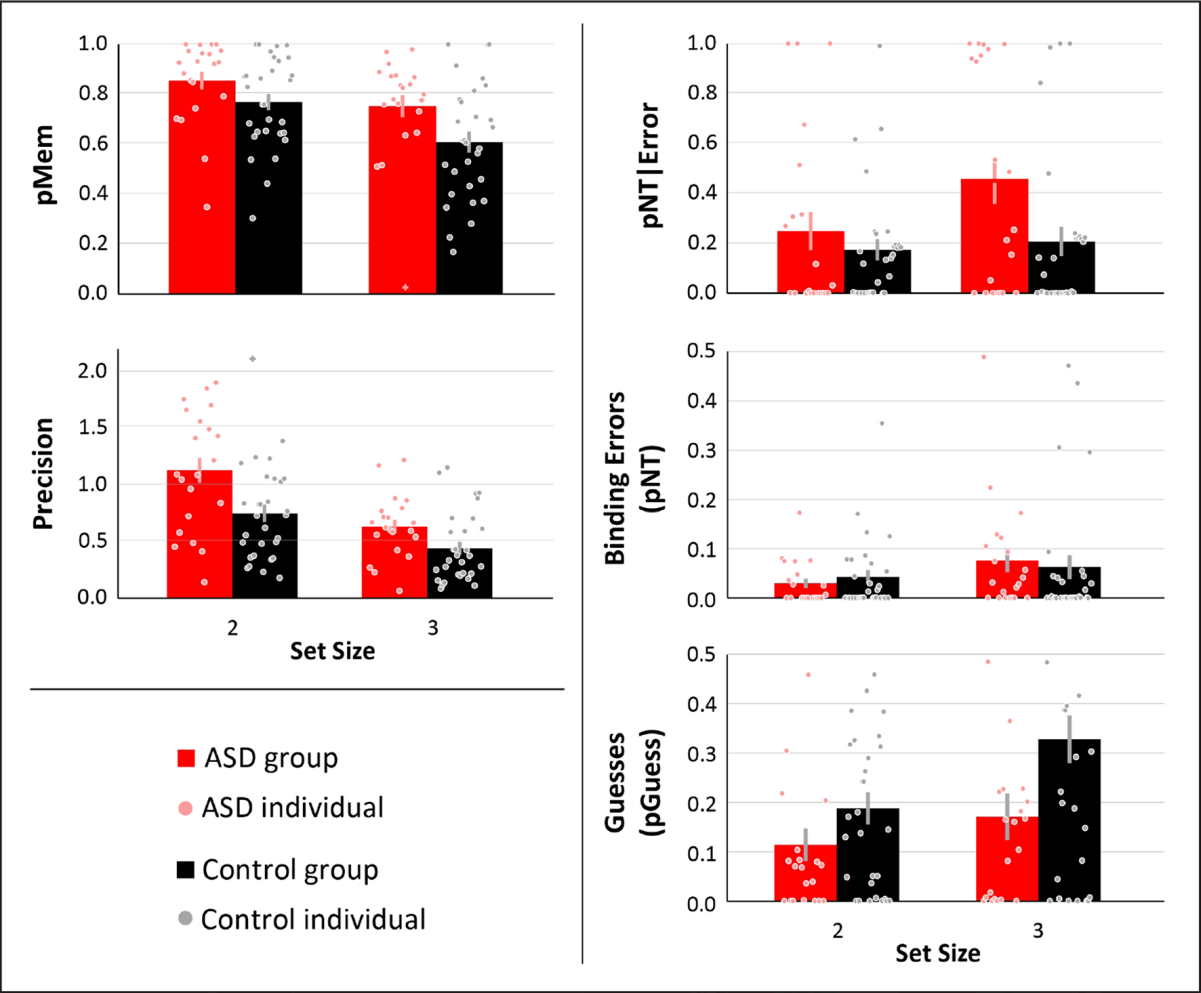
Results. Autistic individuals exhibited increases in their likelihood to remember the target and the precision of their responses, but also an increased NT|Error. Bars represent group averages, circles represent individuals’ data, with crosses representing outliers. Error bars represent standard error of the mean.

We first measured the probability of participants recalling the target colour, or pMem. In the ASD group, pMem was 0.86 (SE = 0.04) for the set size two and 0.75 (SE = 0.05) for set size three (Fig. 2, top). In the TD group, pMem was 0.77 (SE = 0.03) and 0.61 (SE = 0.03) with a set size of two and three, respectively. A 2 × 2 mixed-model ANOVA (group x set size) with pMem as the dependent variable revealed a significant main effect of set size, with the set size of two having a higher pMem than the set size of 3 (F_(1,49)_ = 23.54, p < 0.001, η_p_^2^ = 0.33). Furthermore, there was a significant main effect of diagnostic group, with pMem being higher in the ASD group than the TD group (F_(1,49)_ = 5.19, p = 0.03, η_p_^2^ = 0.10). There was no significant interaction between set size and group (F_(1,49)_ = 1.08, p = 0.30, η_p_^2^ = 0.02).

**Figure 3:**
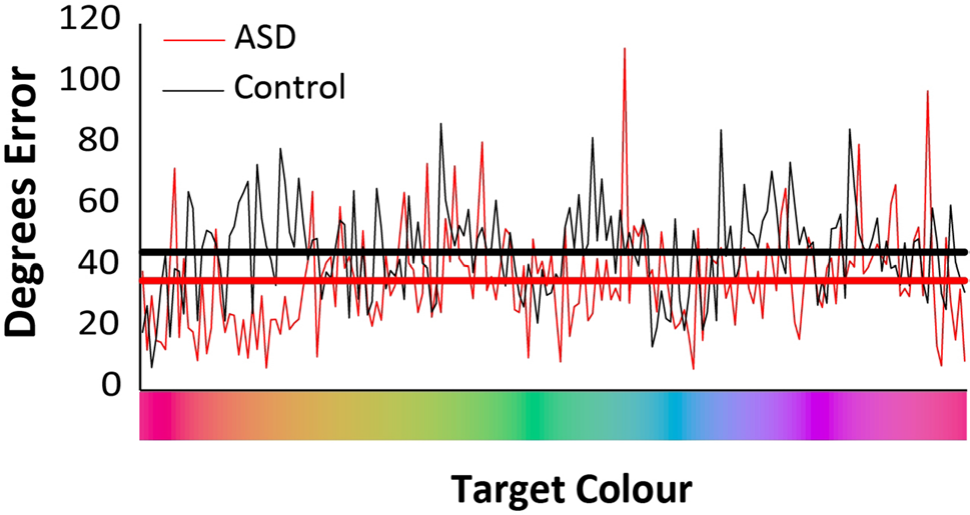
Error by individual colour. Autistic individuals’ higher precision than controls can be seen in their lower average error than controls (heavy lines). No meaningful pattern of error by individual colours was observed (light lines).

Precision was also estimated*,* which reflects the fidelity or resolution with which the colour has been represented in memory, when colour can be retrieved. Precision in the ASD group was 1.13 (SE = 0.12) and 0.63 (SE = 0.06) for set sizes two and three, respectively (Fig. 2, middle). In the TD group, precision was 0.75 (SE = 0.08) and 0.44 (SE = 0.06) for set sizes two and three, respectively. A 2 × 2 mixed-factor ANOVA (group x set size) revealed a significant main effect of set size with higher precision at a set size of two, relative to three, items (F_(1,49)_ = 64.97, p < 0.001, η_p_^2^ = 0.57). Furthermore, there was a significant main effect of diagnostic group, with the ASD group exhibiting higher precision than the TD group (F_(1,49)_ = 7.99, p = 0.007, η_p_^2^ = 0.14). Finally, there was a non-significant trend towards a group by set size interaction; the precision benefit for the ASD group was marginally higher at set size 2 relative to set size 3 (F_(1,49)_ = 3.62, p = 0.07, η_p_^2^ = 0.07). The average error with each presented target colour was also calculated to determine whether precision varied across the colour spectrum in a meaningful, patterned way, and did not (Fig. 3).

### Analysis of error responses: binding errors (pNT) and guesses

When the target was not recalled, two types of errors were possible: specifically, binding errors (pNT) and guesses. Binding errors occur when a participant recalls the non-target colour, and guesses occur when the participant reports a colour that was not presented. To explore the nature of these errors, we estimated the frequency of each type of error, then compared across groups and between conditions in two ways, first accounting for group differences in frequency of errors using pNT|Error, followed by examining values of binding errors and guesses without accounting for the difference in pMem, to examine what drove effects found in the initial analysis.

As reported above, pMem in the ASD group was significantly larger relative to the TD group. This means that the magnitude of binding errors and guesses will, by necessity, be lower in the ASD group. Therefore, we calculated the *proportion* of binding errors relative to total errors, pNT|Error, and subjected those to a 2 × 2 (diagnostic group x set size), mixed-model ANOVA (Fig. 2, top right). A significant main effect of set size was observed, with pNT|Error higher with a set size of three relative to two items (F_(1,49)_ = 4.20, p = 0.04, η_p_^2^ = 0.08). Critically, a main effect of diagnostic group was also observed, with the ASD group exhibiting higher pNT|Error than the TD group (F_(1,49)_ = 4.22, p < 0.04, η_p_^2^ = 0.08). No significant group by set size interaction was observed (F_(1,49)_ = 2.19, p < 0.15, η_p_^2^ = 0.04).

To examine what drove this effect, we examined measures of binding errors and guesses not accounting for differences in pMem. Binding errors in the ASD group were 0.03 (SE = 0.04) and 0.08 (SE = 0.11) for set sizes two and three, respectively (Fig. 2, middle right). In the TD group, binding errors were 0.04 (SE = 0.08) and 0.06 (SE = 0.13) for set sizes two and three, respectively. A 2 × 2 mixed-factor ANOVA (group x set size) revealed a marginally significant main effect of set size with higher binding errors at a set size of three, relative to two, items (F_(1,49)_ = 9.24, p = 0.004, η_p_^2^ = 0.16). There was not a significant main effect of diagnostic group with binding errors (F_(1,49)_ < 0.01, p = 0.99, η_p_^2^ < 0.01). There was not a significant group by set size interaction (F_(1,49)_ = 0.58, p = 0.45, η_p_^2^ = 0.01).

Guesses in the ASD group were 0.11 (SE = 0.03) and 0.17 (SE = 0.05) for set sizes two and three, respectively (Fig. 2, bottom right). In the TD group, guesses were 0.19 (SE = 0.03) and 0.33 (SE = 0.05) for set sizes two and three, respectively. A 2 × 2 mixed-factor ANOVA (group x set size) revealed a significant main effect of set size with higher rate of guessing at a set size of two, relative to three, items (F_(1,49)_ = 9.24, p = 0.004, η_p_^2^ = 0.16). Furthermore, there was a significant main effect of diagnostic group, with the ASD group exhibiting fewer guesses than the TD group (F_(1,49)_ = 5.03, p = 0.03, η_p_^2^ = 0.09). Finally, there was a no significant group by set size interaction (F_(1,49)_ = 1.65, p = 0.21, η_p_^2^ = 0.03).

Given that the TD group exhibited significantly more guesses that the ASD group, it is possible that the increase in pNT|Error was driven by an increase in attentional lapses or failure to encode for other extraneous reasons. We conducted post hoc t-tests to examine this possibility in more detail. No significant difference was found between the ASD and TD group with a set size of two (t_(49)_ = 1.52, p = 0.14, d = 0.43), but a significant difference was observed with a set size of three (t_(49)_ = 2.21, p = 0.03, d = 0.63). No significant difference was found in guesses between set sizes in the ASD group (t_(20)_ = 1.94, p = 0.07, d = 0.42), but a significant increase in guesses was observed from set size 2 to 3 in the TD group (t_(29)_ = 2.80, p = 0.009, d = 0.51).

### Relation to clinical symptomatology

Within the ASD group, multiple regression was used to determine if pMem, precision, or binding errors were associated with symptom severity, as assessed with the SRS and AQ (mean = 0.76, range = 0.57–0.90 of a possible 0–1). The overall model was significant (R^2^ = 0.46, F_(3,17)_ = 4.90, p = 0.01), with pMem being the strongest predictor (t = -3.07, r_partial_ = -0.60, p = 0.007). However, pMem and precision were multicollinear (r_20_ = 0.84, 95% CI = [0.64 to 0.55], VIF = 3.35, p = 0.0004). For thoroughness, it should be noted that binding errors were not related to either pMem (r_20_ = 0.01, 95% CI = [-0.42 to 0.44], p = 0.96) or precision (r_20_ = 0.32, 95% CI = [-0.12 to 0.66], p = 0.16). Given the multicollinearity, precision was removed from the model, and a second multiple regression was conducted using pMem and pNT|Error to predict symptom severity. The overall model remained significant (R^2^ = 0.40, F_(2,18)_ = 6.00, p = 0.01), with pMem (t = -3.46, r_partial_ = -0.63, p = 0.003) but not pNT|Error (t_(20)_ = 1.28, r_partial_ = 0.29, p = 0.22) significantly predicting symptom severity.

### Sex differences

Given the differences in sex distribution across groups, sex differences were explored. No significant differences were found between males and females in pertinent variables at either set size (2 and 3, respectively), including pMem (t_(49)_ = 1.15, p = 0.11, *d* = 0.32; t_(49)_ = 0.23, p = 0.90, *d* = 0.06), precision (t_(49)_ = 0.81, p = 0.59, *d* = 0.23; t_(49)_ = 0.55, p = 0.34, *d* = 0.15), binding errors (t_(49)_ = 1.44, p = 0.07, *d* = 0.40; t_(49)_ = 1.52, p = 0.07, *d* = 0.42), pNT|Error (t_(49)_ = 0.55, p = 0.48, *d* = 0.15; t_(49)_ = 0.88, p = 0.14, *d* = 0.25), or guesses (t_(49)_ = 0.66, p = 0.18, *d* = 0.; t_(49)_ = 0.50, p = 0.85, *d* = 0.14).

## Discussion

Through the use of a well-established VWM continuous cued-recall task, this study provided novel evidence reconciling two apparently discrepant findings relating to sensory processing in autism. That is, both enhanced and impaired sensory processing in autism have been shown with almost equal frequency for review, see^14,19^. Typically, these differences have been shown across distinct tasks^37^ and stimulus types^38^, with enhanced perception commonly found with simple stimuli and detail-oriented tasks and impaired perception commonly seen with more complex stimuli and globally-oriented tasks. Results from the current study, for the first time, indicate enhanced sensory processing in both accuracy and precision of sensory recall, yet increased binding errors when errors are made: distinct aspects of sensory processing using the same task, stimuli, and individuals.

The Enhanced Perceptual Functioning (EPF) model of sensory processing in autism proposes that autistic individuals prioritize the perception of low-level sensory information relative to higher-level perceptual operations^15,19,39^. This difference in processing has been hypothesized to lead to both enhancements in perceptual abilities (e.g., enhanced pitch memory^40^ and visual discrimination^41^), but differential abilities at higher levels of perception (e.g., biological motion perception^42^)—a combination of perceptual effects commonly referred to as a difference in local–global bias. This account has typically been described through comparing performances across different cognitive tasks or stimuli. For example, when processing composite letters, autistic individuals show a bias towards over-representation of the smaller, component letters relative to their TD peers, but this effect disappears when specifically asked to attend to the larger, global letters^37^. Thus, it has been postulated that deficits in more global, integrative sensory processing may result from a difference in default perceptual style, with a tendency to focus on the detailed aspects of any given sensory input. The current results expand on this finding, however, suggesting that findings of perceptual enhancements and differences in autism are not specifically task-reliant but instead process-reliant, as can be observed simultaneously in a single paradigm.

Autistic children exhibited specific perceptual advantages in their ability to recall visual representations (as measured by pMem), and additionally, the *precision* of those representations was of higher fidelity then their TD peers. That is, when correctly remembering a colour in a specific location, autistic children’s ability to recall the exact hue of the colour was greater. Interestingly, recent computational work has suggested that both precision and pMem may be accounted for by a single factor of memory strength, or sensitivity^43^—and explanation that is parsimonious with the current data given the strong relationship between pMem and precision. Interestingly, the perceptual advantage of increased likelihood of recall significantly was predictive of clinical symptom severity as assessed through the Social Responsiveness Scale and the Autism-spectrum Quotient. This relationship between levels of autistic traits and performance in a VWM task provides convergent evidence that atypical sensory processing may contribute to clinical symptomatology for review, see^14^.

The analysis of concurrent error types was more equivocal, with multiple possible interpretations. When examining binding errors relative to the total proportion of errors committed (pNT|Error), autistic participants committed a higher proportion of *binding errors*; they incorrectly perceptually bound a presented colour with the wrong spatial location. In conjunction with the observed increase in *precision*, this would suggest that the perceptual representation of colour is being maintained with high fidelity. Further, given the higher level of pMem in ASD relative to controls, this suggests that autistic individuals broadly have the capacity to bind colour and space. However, the autistic group showed a higher proportion of binging errors relative to the total number of errors than the control group. This finding is in line with previous research, which has reliably demonstrated that autistic individuals exhibit atypical integration of individual pieces of information to form a coherent Gestalt percept^20–22^. This effect ranges from the perceptual processing of sensory stimuli such as a speaker’s face and voice^24–27^ to higher-level cognitive representations, such integrating content of a story into a global narrative^44^.

The above hypothesis of atypical binding reflects the rate of binding errors *when an error is committed.* This phenomenological finding has multiple possible alternative explanations at the mechanistic level. The increase in pNT|Error in the autistic group was driven by a significantly higher rate of guesses in the control group, in particular with a set size of three. Thus, alternatively to the above hypothesis, analysis of the binding errors without accounting for group differences in pMem did not reveal group differences, leaving the possibility that there a greater number of attentional lapses in the control group may also contribute to or drive this effect. Further, if autistic children were inherently better at color than spatial attention (within ASD), or if autistic children were better at colour memory than controls, this could result in performance in the autistic group to be biased towards colour rather than space more so than the control group. Lastly, the autistic group may be more likely to focus on the task, and, as a colour memory task, may have biased the autistic group more than the control group towards attending to colour. Future studies in which the task was to remember the location will be able to assess these alternate hypotheses.

How might we understand the present pattern of results from a neuro-computational perspective? Recent computational models have proposed that both precision and binding errors in VWM can be explained through decoding of noisy population neural activity^45,46^. Specifically, the model consists of populations of neurons, which respond conjunctively to multiple feature dimensions (e.g., color, orientation, location). For example, a given neuron in the model might respond most strongly to a specific hue of green, at a specific location, with the firing rate decreasing as the presented hue (or location) deviates from the maximally preferred value. In this way, the firing properties of each neuron can be represented as a multivariate Gaussian tuning function across all feature dimensions of interest. During study, sample items are encoded and maintained through the firing rate of the neural populations, which are determined by their respective tuning preferences. During recall, a decoder is applied to the pattern of population activity, ultimately selecting the most likely feature value, given the cue. Thus, the imprecision of responses can arise from population neural noise across the target dimension (e.g., colour), while binding errors arise from noise across the cue dimension (e.g., location). Applied to our data, this model would suggest that autistic individuals represent sample items with *lower* levels of noise in the target dimension, but that *higher* levels of noise in the cue dimension result in an unreliable mapping of the location cue to the correct color, thereby resulting in binding errors between colors and their locations. Indeed, autism researchers have suggested that atypical behaviours and perceptions in autism may be linked to changes in the variability, or noisiness, of neural response patterns^47–51^.

Based on their work with healthy populations, Bays et al.^45,46^ also argue that in VWM recall tasks, non-spatial features (e.g., color and orientation) are obligatorily bound to their associated locations, with no binding between non-spatial features. Similarly, face perception research suggests that the healthy human visual system automatically binds facial elements into an undifferentiated whole^52^. Given the deficits in holistic face perception in ASD^53,54^, along with group differences in binding deficits between a spatial and non-spatial feature when accounting for differences in overall error rates reported here, an interesting prediction is that autistic individuals might only show binding difficulties across features that are obligatorily integrated in the healthy visual system.

Additionally, integration of multiple types of sensory information, in this case spatial and colour information, requires functional connectivity between multiple brain regions. However, research has demonstrated that autistic individuals have decreased connectivity between brain regions in general^55,56^, and within the dorsal visual stream specifically^57^. Furthermore, autistic individuals show atypical activation, functional connectivity, and inter-region neural synchronization in fronto-parietal networks during letter-based VMW tasks^58^ and in fronto-temporal networks during face-based VWM tasks^59^. Coupled with the current findings, these studies lend support to the claim of the EFT model suggesting that improved processing of individual aspects of sensory inputs may come with a cost, in this case including higher levels of binding errors relative to all errors during VWM. These changes are likely to directly impact core symptomatology associated with autism, as perceptual binding^60^ and working memory, including VWM^61^, play significant roles in communication.
